# Royal National Life-Boat Institution

**Published:** 1863-12

**Authors:** 


### ROYAL NATIONAL LIFE-BOAT INSTITUTION.

### DIRECTIONS FOR RESTORING THE APPARENTLY DROWNED.

From London Lancet.

The leading principles of the following Instructions are those of the late Dr. Marshall Hall, for the Restoration of the apparently Dead from Drowning, and are the results of the latest discoveries:

Send immediately for medical assistance, blankets, and dry clothing; but proceed to treat the patient *instantly* on the spot, in the open air, whether on shore or afloat.

The points to be aimed at are, *first* and *immediately*, the restoration of breathing and the prevention of any further diminution of the warmth of the body; and secondly, after breathing is restored, the promotion of warmth and circulation.

The efforts to restore breathing, and to prevent any further diminution of the warmth of the body, must be commenced immediately and energetically, and must be persevered in for several hours, or until a medical man has pronounced that life is extinct. Efforts to promote warmth and circulation must be deferred until natural breathing has been restored.

To Restore Breathing—To Clear the Throat. 1—Place the patient on the floor or ground, with his face downwards, and one of his arms under the forehead, in which position all fluids will escape by the mouth, and the tongue itself will fall forward, leaving the entrance into the windpipe free. Assist this operation by wiping and cleansing the mouth.

2—If satisfactory breathing commences, adopt the treatment described on the next page to promote warmth and natural breathing. If there be only slight breathing, or no breathing, or if it fail, then

To Excite Breathing. 3—Turn the patient well and instantly on the side and—

4—Excite the nostrils with snuff, hartshorn, and smelling salts, or tickle the throat with a feather, etc., if they are at hand. Rub the chest and face warm, and dash cold water on it.

5—If there be no success, lose not a moment, but instantly

To Imitate Breathing. 6—Replace the patient on the face, raising and supporting the chest well on a folded coat or other article of dress.

7—Turn the body very gently on the side and a little beyond, and then briskly on the face, back again; repeating these measures deliberately, efficiently and perseveringly about fifteen times in the minute, or once every four seconds, occasionally varying the side: (By placing the patient on the chest, the weight of the body forces the air out; when turned on the side, this pressure is removed, and the air enters the chest.)

8—On each occasion that the body is replaced on the face, make uniform but efficient pressure, with brisk movement, on the back between and below the shoulder-blades or bones on each side, removing the pressure immediately before turning the body on the side: (The first measure increases the expiration, the second commences inspiration.)

*** The result is respiration or natural breathing; and if not too late, life.

Cautions. 1—Be particularly careful to prevent persons crowding round the body.

2—Avoid all rough usage and turning the body on the back.

3—Under no circumstances hold the body up by the feet.

To Prevent any further Diminution of Warmth.—These efforts must be made very cautiously, and must not be such as to promote *warmth* and *circulation rapidly;* for if circulation is induced before breathing has been restored, the life of the patient will be endangered. No other effect, therefore, should be sought from them than the prevention of evaporation, and its result, the diminution of the warmth of the body.

1—Expose the face, neck, and chest, except in severe weather, (such as heavy rain, frost, or snow.)

2—Dry the face, neck, and chest as soon as possible with handkerchiefs or anything at hand; and then dry the hands and feet.

3—As soon as a blanket or other covering can be obtained, strip the body; but if no covering can be immediately procured, take dry clothing from the bystanders, dry and reclothe the body, taking care not to interfere with the efforts to restore breathing.

CAUTIONS. 1—Do *not* roll the body on casks.

2—Do *not* rub the body with salt or spirits.

3—Do *not* inject tobacco-smoke or infusion of tobacco.

4—Do *not* place the patient in a warm bath.

